# Cholera during COVID-19: The forgotten threat for forcibly displaced populations

**DOI:** 10.1016/j.eclinm.2021.100753

**Published:** 2021-02-11

**Authors:** Osama B Hassan, Laura B Nellums

**Affiliations:** Division of Epidemiology and Public Health, School of Medicine, University of Nottingham UK

Cholera has been a global public health challenge since 1817. This acute, diarrhoeal, infection caused by the bacterium *Vibrio cholerae,* is transmitted by the faecal-oral route through contaminated water or food. Cholera annually affects 2.9 million people, causing 95,000 deaths worldwide. Forcibly displaced populations experience high rates of cholera due to limited access to healthcare and poor living conditions, including overcrowding, and disruption of water, sanitation, and hygiene (WASH) services. Cholera is preventable and predictable; however, it can be fatal without proper and timely response.

The increased threat of cholera should not be overlooked at this time when government and non-governmental organization (NGO) health services are overwhelmed by COVID-19. Ethiopia's Tigray crisis provides a critical example of the heightened risk of cholera posed by the intersection of the pandemic and escalating violence; food and water shortages; displacement; and disruptions to infrastructure and health systems.

Both Ethiopia and Sudan are cholera endemic regions, which have experienced several cholera epidemics, including recent outbreaks in 2019, 2020, and 2021 [1,2]. In Ethiopia, the current COVID-19 pandemic and insecure regions in the country have halted the response to the ongoing cholera epidemic, which has caused around 15,000 cases and 250 deaths [2,3].

The conflict in the Tigray region puts 2.3 million people at risk, with immediate needs and limited humanitarian access [4]. Since the conflict erupted, 53,302 Ethiopian refugees have entered Sudan, staying in temporary shelters and Um Raquba refugee camp, which is reaching maximum capacity [4]. Concerns of a severe humanitarian crisis have been expressed due to lack of shelter, food, medical supplies, and WASH services [4]. This coincides with the economic strains, lack of resources, and limited health system infrastructure Sudan and humanitarian organisations are experiencing during COVID-19.

Early prediction and preparedness are pivotal to avoid tragic cholera outbreaks like the current one in Yemen, which started in 2016, and has caused over two million suspected cases and nearly 4000 deaths [5]. Prevention and control of infectious diseases in displaced populations requires a comprehensive, multidisciplinary, collaborative approach. The early implementation of proper surveillance systems, through training of health workers on testing and reporting, along with improving health infrastructure to facilitate access to healthcare, are highly recommended to control infections in emergency settings. Oral cholera vaccination has also proved to be feasible and effective in reducing risk of cholera infection in refugee settings [6]. This would also pave the way for possible COVID-19 vaccinations of displaced populations.

Improving living conditions and provision of proper and sustainable WASH services constitutes a long-term approach in infection control. This involves provision of adequate shelter to reduce overcrowding; supplying food, and good quality and quantity of water supply; building sufficient latrines with proper sewage systems; and distributing enough hygiene kits in order to promote personal hygiene behaviours such as hand washing. Health education and raising awareness, supported by meaningful engagement of the affected communities, is also important to enhance resilience, ownership, and uptake of response measures to prevent and control cholera, COVID-19, and other communicable diseases. These measures are emphasised in the Global Task Force on Cholera Control eradication strategy 2030 to reduce cholera death by 90%, and must be prioritised in forced migrant contexts given evidence that attack rates and case fatality rates may be elevated [7]. We have an opportunity to harness the momentum of COVID-19 to strengthen infection prevention and control, tackling the pandemic, as well as ongoing threats, which must not be forgotten.

## Author contribution

Both authors were involved in the conception and writing of this manuscript.

## Declaration of Competing Interest

None to declare.
